# Capturing the subject-specific quality of mathematics instruction: How do expert judgments relate to students’ assessments of the quality of their own learning and understanding?

**DOI:** 10.1007/s11858-024-01561-3

**Published:** 2024-03-20

**Authors:** Christine Pauli, Frank Lipowsky, Kurt Reusser

**Affiliations:** 1 University of Fribourg, Fribourg, Switzerland; 2 University of Kassel, Kassel, Germany; 3 University of Zurich, Zurich, Switzerland

**Keywords:** Subject-specific teaching quality, Students’ perceptions of their own learning process, Mathematics instruction, Opportunity-use models

## Abstract

Based on an opportunity-use model of instructional quality, this study investigates the extent to which subject-specific instructional quality rated by experts is reflected in students’ assessments of their own learning and understanding, and how students’ perceptions predict their achievement. The analyses used data from a German-Swiss sample of 36 classes with around 900 lower secondary students, obtained as part of the so-called “Pythagoras study” in the school year 2002/2003. The teachers were instructed to introduce the Pythagorean theorem in three lessons, which were videotaped. Using the videos, the experts assessed the instruction quality with respect to the goal of promoting a deep understanding of the theorem. The students completed the questionnaires assessing their understanding of the content, their learning process, and the general comprehension orientation of the teacher. The results showed significant and moderate correlations on the class level between expert-rated subject-specific teaching quality and students’ perceptions of their own learning and understanding, as well as of the teacher’s general comprehension orientation. Multilevel models revealed that subject-specific expert ratings are reflected in individual students’ perceptions of their own learning and understanding. Student perceptions were also associated with achievement gains. The results suggest that the assessment of quality by students and experts is more closely linked if a distinction is made between the quality of the learning opportunities offered and their use and if subject-specific criteria are used instead of generic criteria. This study contributes to a more nuanced understanding of the validity of student perspective in assessing instructional quality.

**Supplementary Information:**

The online version contains supplementary material available at 10.1007/s11858-024-01561-3.

## Introduction

Cognitive-constructivist theories of learning emphasize students’ active role in learning with respect to the goal of deep and robust understanding (Brunner, 2018; Chi & Wylie, 2014; Koedinger et al., 2012; Reusser, 2006). This is also reflected in “opportunity-use-models” of teaching and learning (Fend, 1998; Helmke, 2003; Reusser & Pauli, 2010; Vieluf & Klieme, 2023). According to these models, students’ learning processes cannot be controlled from outside; rather, their learning depends on them making the most effective use of the learning opportunities offered by the teacher. This suggests that student perspective should also be considered when examining teaching quality. Students’ perceptions have been included in various studies on teaching quality and, in some cases, compared with teacher or expert ratings (e.g. Cheng et al., 2023; Clausen, 2002; Fauth et al., 2020; Göllner et al., 2021; Kunter & Baumert, 2006; Wang & Eccles, 2016). Most of these studies have revealed surprisingly low correlations between expert and student perspectives. Apparently, students are rather poor at assessing deep structural features of instructional quality.

However, there are two limitations to the abovementioned research. First, the students were mostly asked about their perceptions of the learning opportunities and not about their use of these opportunities. We assume that students are quite able to assess important aspects of instructional quality when asked about their own learning and understanding processes (Jansen et al., 2022; Merk et al., 2021). Second, the expert and student ratings used in these studies were mostly related to the generic aspects of teaching quality, although subject-specific aspects seem to be particularly important for promoting a deep and robust understanding of concepts (cf. Drollinger-Vetter, 2011; Hiebert & Grouws, 2007; Schlesinger & Jentsch, 2016; Schlesinger et al., 2018; Schoenfeld, 2018).

The analyses presented here address both aspects. We investigate the question of how expert and student judgments are related when expert judgments refer to subject-specific quality characteristics of teaching and student judgments refer to the use of these learning opportunities, as well as the relation between student judgments and learning progress. The present analyses draw on the data and earlier analyses in the context of the so-called “Pythagoras study” (Klieme et al., 2009; Rakoczy et al., 2007). The database generated in this study facilitates a comparison of perspectives, considering both the opportunity-use model and subject-specific quality characteristics, even though the data was already obtained in 2002 (see 6.2 for a discussion).

## Theoretical framework

### Teaching toward understanding–a crucial feature of instructional quality

Enabling and fostering deep conceptual understanding is a central goal for all students not only in mathematics instruction but in all school subjects (Gravemeijer et al., 2017; Patrick et al., 2012; Reusser & Reusser-Weyeneth, 1997). Understanding content means mindfully realizing the inner relations of concepts, i.e. recognizing the connections between and within concepts, and using them for generating new ideas and thoughts (e.g. Aebli, 1980/1981; Drollinger-Vetter, 2011; Koedinger et al., 2012; Wertheimer, 1945). From a cognitive-constructivist perspective, the development of thoroughly understood mathematical knowledge by students requires a focused and intensive cognitive processing of the learning contents in terms of deep learning. This includes using prior knowledge, establishing connections between existing and new knowledge, constructing mental models, and integrating newly created knowledge into one’s own knowledge base through constructive cognitive activities (Hiebert & Carpenter, 1992; Schlesinger & Jentsch, 2016).

For comprehension-oriented mathematics teaching, the challenge is to initiate, instruct, and support students in these processes. It has become increasingly clear that comprehension-orientated instruction depends less on specific methods than on deep structural features, such as the extent to which students’ cognitive engagement is stimulated and supported as they engage with the learning content (Hiebert & Grouws, 2007; Mayer, 2009; Praetorius & Charalambous, 2018; Prediger et al., 2022; Reusser, 2005).

### From generic to subject-specific concepts of teaching quality: challenges of measurement with a focus on comprehension

In recent years, the deep structural quality of teaching has often been measured using a three-dimensional framework. It is based on the three dimensions of cognitive activation, student support, and classroom management (Klieme et al., 2009; Kunter & Voss, 2013), which are also referred to as “three basic dimensions” (TBDs) of teaching quality. Both observation protocols (Lipowsky et al., 2009; Praetorius et al., 2018) and student questionnaires (e.g. Herbert et al., 2022; Senden et al., 2023) were developed on the basis of the TBDs. The “cognitive activation” dimension is particularly important for assessing comprehension orientation, as it focuses on the extent and quality of students’ cognitive activities. Observation protocols record, for example, the quality of the tasks being worked on or certain characteristics of classroom interactions (cf. Praetorius et al., 2018).

Most of these instruments capture “cognitive activation” as a generic feature of instructional quality that does not focus on specific subjects or content. However, there is a growing consensus that teaching quality also includes subject-specific aspects. It is not a matter of creating as *many* connections as possible but of recognizing and elaborating the elements and relationships that are *relevant* for the understanding of a concept in a transparent and coherent development process. Comprehension-oriented mathematics instruction helps learners focus on essential principles and conceptual elements, and links them through active, in-depth processing into correct and coherent subject-specific knowledge and thought structures (e.g. Hiebert & Grouws, 2007; Prediger et al., 2022). For (research on) teaching, this demands a subject-specific analysis of the content-related pedagogical requirements for understanding a particular concept or content structure (Drollinger-Vetter, 2011; Schlesinger & Jentsch, 2016). In fact, the call for greater consideration of the subject-specific perspective on teaching quality has gained prominence in recent years (e.g. Brunner, 2018; Dreher & Leuders, 2021; Lindmeier & Heinze, 2020; Schlesinger et al., 2018).

#### Measuring instructional quality with consideration for opportunity-use models

Recently, various observation protocols have been developed that aim to capture the subject-related pedagogical aspects of the quality of mathematics lessons (for comprehensive overviews, see e.g., Charalambous & Praetorius, 2018; Schlesinger & Jentsch, 2016). What these instruments have in common is that their application requires a high level of expertise in mathematics as well as in the didactics of mathematics (Dreher & Leuders, 2021). Therefore, it is not surprising that studies on the subject-specific quality of mathematics teaching have so far relied primarily on expert ratings.

Based on opportunity-use models that view teaching quality as an interplay between the provision and use of learning opportunities (Vieluf & Klieme, 2023), the assessment should not only focus on the teacher’s providing behavior but also include the students’ use of it. It can be assumed that observers’ judgments of quality not only refer to features of teachers’ tasks and behavior but are also likely to include the perceived features of student behavior to some extent (Fauth et al., 2020). Thus, when assessing cognitive activation, it can be assumed that the signs of students’ cognitive activity or attention are partially considered, and conclusions are drawn about the quality of students’ understanding and learning processes. Global assessments, however, only partially capture how individual students use the offering based on their learning prerequisites. One possible solution is to include student perceptions.

#### Measuring instructional quality from students’ viewpoints

For years, it has been a common approach in classroom research to measure instruction and its quality from students’ viewpoints (e.g. De Jong & Westerhof, 2001; Wagner et al., 2013). However, only a few studies have systematically differentiated between the perception of the learning opportunities offered (e.g., tasks and teacher behavior) and their use (e.g., cognitive engagement and comprehension).

The validity of student ratings of instructional quality has been extensively studied (e.g. Clausen, 2002; Herbert et al., 2022; Kunter & Baumert, 2006; Lenske & Praetorius, 2020; Scherer et al., 2016; Senden et al., 2023; Wagner et al., 2013; Wisniewski et al., 2020). Overall, the results on the *discriminant validity* of such student judgments show that students can discriminate among different dimensions of teaching quality, including those of the TBDs (e.g. Fauth et al., 2014; Senden et al., 2023). Students’ perceptions of teaching quality have certain *prognostic validity* for cognitive, motivational, and social student outcomes. Overall, the empirically observed associations between students’ perceptions of teaching quality and achievement gains are rather small and concern classroom management rather than cognitive activation (Herbert et al., 2022; Kunter & Baumert, 2006; Scherer et al., 2016; Senden et al., 2023; Waldis et al., 2010; Wallace et al., 2016). In terms of *convergent validity*, comparisons of student perceptions with expert or teacher ratings show significant relations if the ratings refer to well-observable characteristics of instruction and to the social aspects of learning, but–with a few exceptions (e.g. Cheng et al., 2023)–no or less pronounced agreement for characteristics such as cognitive activation and learning support (e.g. De Jong & Westerhof, 2001; Kunter & Baumert, 2006).

As a possible explanation for the rather low correlations between student and observer judgments, Lenske and Praetorius (2020) showed that the students sometimes misunderstood the items of an assessment questionnaire. This was especially the case for “cognitive activation.” In addition, rating scales that focus on instructional quality sometimes contain a mix of items that ask about perceptions of instructional features and items that focus on student behaviors (see also Fauth et al., 2020).

#### Revisiting the measurement of instructional quality from students’ perspectives as a desideratum of research

Note that the student questionnaires used in most of the above studies did not record teaching quality in a subject-specific manner and were primarily aimed at the perception of learning opportunities and not their use. Even though several instruments for observer ratings of subject-specific quality characteristics have been developed recently (see 2.2.1), only a few studies have surveyed both expert and student perceptions in connection with subject-specific quality features of comprehension-oriented teaching (e.g. Cheng et al., 2023; Scherer & Gustafsson, 2015). This is understandable, as an assessment of subject-specific quality characteristics requires subject-specific and didactic expertise. Based on an opportunity-use model, it makes sense to ask students about the *use* of learning opportunities and not about the subject-specific characteristics of these opportunities. The latter can be better assessed by experts. Students’ perceptions of their own understanding and learning can show how they use the offer (Jansen et al., 2022; Merk et al., 2021; Rieser & Decristan, 2023; Vieluf, 2022).

So far, little is known about how closely students’ assessments of their own learning and comprehension processes are related to expert assessments that focus on subject-specific features of comprehension-oriented mathematics instruction. Earlier analyses in the context of the Pythagoras study, which addressed a different question and included only one aspect of subject-specific teaching quality, have already indicated such a relation (Rakoczy et al., 2007). Using the same database, the present study examines the extent to which experts’ subject-specific quality judgments are reflected in students’ perceptions of their own learning and understanding and the teacher’s general orientation toward understanding, as well as the extent to which students’ self-assessments predict their learning gains. The aim is to contribute to the investigation of the convergent and prognostic validity of student perceptions considering both subject-and content-related characteristics of teaching quality and an opportunity-use model.

## Research questions

The following research questions (RQs) are addressed:

- 1a) Are expert ratings of subject-specific quality features of instruction related to students’ (class-level) perceptions of their own learning process and content understanding when both students and experts refer to the same mathematics lessons?
- 1b) Are expert ratings of subject-specific quality features of instruction related to students’ (class-level) ratings of the teacher’s overall comprehension orientation (as measured at the end of the school year)?
- 2a) Are expert ratings of subject-specific quality features of instruction related to individual students’ perceptions of their own learning process and content understanding when both relate to the same mathematics lessons?
- 2b) Are expert ratings of subject-specific quality features of instruction related to individual student perceptions of the teacher’s overall comprehension orientation?
- 3) Are individual student perceptions of their own learning and understanding and of the teacher’s overall understanding orientation related to learning success when central learning prerequisites are controlled for?

## Method

### Database

The present study is embedded in the quasi-experimental project “Quality of instruction, learning, and mathematical understanding” (also called the “Pythagoras study”), which investigated the impact of mathematics instruction on students’ cognitive and motivational outcomes over the period of one school year (Hugener et al., 2009; Klieme et al., 2009; Lipowsky et al., 2009). In particular, the present analysis extends previous analyses that investigated the cognitive and motivational effects of structured instruction (Rakoczy et al., 2007). The original sample comprised 20 Swiss and 20 German classes with 1015 students from two lower secondary school types: the highest track (*Gymnasium)* and the intermediate track (*Realschule, Sekundarschule*).^1^ Participation was voluntary. The present analyses draw on data from a reduced sample comprising 36 classes^2^ and a maximum of 913 students. In all classes, a three-lesson unit on the “Introduction to Pythagorean Theorem” and a two-lesson unit on algebraic word problems were videotaped in the school year 2002/2003.

### Study design

The analyses presented are based on the “Pythagorean unit.” The teachers were asked to submit at least one proof (of any kind) of the Pythagorean theorem; otherwise, they were free to organize the lessons as they wished. Data were collected at four measurement time points. At the beginning of the school year, the students’ general mathematics achievement and cognitive ability were tested. In addition, their characteristics such as interest in mathematics were assessed using a questionnaire (T1). Immediately before the three-lesson unit “Introduction to the Pythagorean Theorem,” the students were tested on their prior knowledge of the Pythagorean theorem (T2). Immediately after the three lessons, the students assessed their learning and understanding (questionnaire, T3). Subsequently, learning success was assessed (post-test Pythagoras, T3). At the end of the school year, the students’ overall mathematics achievement was tested again, and their general perceptions of mathematics instruction and the mathematics teacher were recorded (T4).

### Measures of students’ perceptions

The scale items can be found in the Appendix online (“Supplementary Information”). The instruments used were developed as part of the Pythagoras study.

#### Scale “students’ perceptions of their learning process”

The students’ self-reported cognitive processes during the three Pythagoras lessons were assessed at T3 using four items (4-point-response scale, items: see Appendix). The reliability coefficient (Cronbach’s alpha) was 0.86, the mean of the scale was 3.34, and the standard deviation was 0.58 (Rakoczy et al., 2005).

#### Single item “students’ perception of their own attained Pythagoras-related understanding”

The students’ perceptions of their own attained understanding were assessed at T3 using the single item “How well have you understood the content that you went through?” The students responded to this question on a six-point scale (see Appendix). The mean of the item was 5.07, and the standard deviation was 0.97 (Rakoczy et al., 2005). The higher the value, the higher the students rated their comprehension.

#### Scale “students’ perceptions of the overall comprehension orientation of the math teacher”

The students’ perceptions of the comprehension orientation of the teacher and his/her teaching were assessed at T4 (end of the school year) using a bipolar 4-item scale. The students had to decide which pole best represented their opinion (six-point response scale, see Appendix). The higher the value, the higher the students estimated the teacher’s focus on understanding. The reliability was *α* = 0.86, and the mean of the scale was *M* = 4.67 (*SD* = 1.08).

Correlations were found among the three measures (Table A1, Appendix). The association was stronger when both perceptions referred to the Pythagorean unit (*r* = 0.66**). In contrast, the correlations were lower if one of the two variables related to the teachers’ overall comprehension orientation and thus to the long-term period of the school year (*r* = 0.22**; *r* = 0.11**).

#### Calculation of ICC(1) and ICC(2)

Beyond the individual perceptions of the students, aggregated student judgments can be used as a source of information about the comprehension orientation of the lessons and the teacher as characteristics of the shared environment. For this, it is necessary to calculate *ICC*(1) and *ICC*(2) (Lüdtke et al., 2009). The results for *ICC*(1) revealed that between 9 and 15% of the variance in student perceptions could be explained by belonging to the class; consequently, there were substantial differences between the classes. The values for *ICC*(2) lied above 0.70 or just below (Table A2, Appendix). Therefore, it can be assumed that student judgments reflect differences between the learning conditions in classes and not idiosyncratic perceptions.

### Measures of students’ achievements, mathematics-related interest, and general cognitive abilities

#### Mathematical achievement

The data from all achievement tests were scaled using the ConQuest program (Wu et al., 1997), based on a one-parameter item-response model (Rasch model; for details see Lipowsky et al., 2006). The four achievement tests used were scaled independently of each other.

At T1, we measured the general mathematics knowledge with 10 items regarding basic skills, understanding mathematical proofs, and application ability. The EAP/PV reliability was 0.60, and the weighted mean-square residuals (MSQs) were between 0.96 and 1.08.

The students’ mathematics achievement before and after the Pythagorean theorem was measured in a content-specific manner. The Pythagorean pre-test (T2) focused on the major prerequisites for a conceptual understanding of the Pythagorean theorem. Individual achievement scores were estimated separately for the pre-test and post-test using item response theory. For the pre-test, the mean square parameters of the items ranged between 0.92 and 1.03; the EAP/PV reliability was 0.64.

The Pythagorean post-test (T3) focused on the conceptual understanding of the Pythagorean theorem and its application to simple tasks. The post-test took additional 15 min to complete. The mean square parameters of the items ranged between 0.89 and 1.24; the EAP/PV reliability was 0.78.

At T4, we measured the general knowledge with 18 items referring to basic skills, algebra skills, and application ability. The EAP/PV reliability score was 0.72, and the weighted MSQs were between 0.91 and 1.09.

#### Scale “mathematics-related interest”

Interest in mathematics was recorded at T1 using 8 items in the student questionnaire (Appendix). Answers were recorded on a four-point scale. The reliability coefficient (Cronbach’s alpha) was 0.91, the mean of the scale was 2.69, and the standard deviation was 0.71 (Lipowsky et al., 2009; Rakoczy et al., 2005).

#### General cognitive ability test

The students’ general cognitive abilities were measured at T1 using a subtest of the Heller and Perleth (2000) cognitive abilities test. The mean amounted to 50.98 points, and the standard deviation was 9.96 points. This is in line with the mean of 50 points and the standard deviation of 10 points for the *t*-scale (Lipowsky et al., 2009).

### Expert ratings of subject-specific instructional quality

Two experts–a co-author and an expert in mathematics education–assessed the subject-specific quality of the videotaped lessons using a rating protocol that they had jointly developed under the leadership of the mathematics education expert. They focused on the theory and proof phases of the lessons in which concepts were introduced, theorems were stated, and proofs were given (Drollinger-Vetter, 2011, p. 227; Drollinger-Vetter et al., 2006, Appendix). To comprehensively assess the subject-specific quality, they focused on the occurrence of conceptual elements (“elements of understanding”: EoU), the quality of modes of representation, and the structural clarity of the content covered in the Pythagoras lessons (Drollinger-Vetter & Lipowsky, 2006).

#### Score “occurrence of EoU”

The experts analyzed the content frame of the Pythagorean theorem by asking what conceptual elements (EoU) of the Pythagorean theorem a teacher should address in the introductory instruction to promote students’ sustained understanding of this content (Drollinger-Vetter, 2011). Nine EoU were identified as essential for a deeper understanding of the Pythagorean theorem (see Appendix). The experts rated whether these EoU were treated in the three-lesson unit and, if so, whether it was extensive or short. Each EoU was scored with 1 (= no occurrence), 2 (= short occurrence), or 3 (= extensive occurrence).

The occurrence of the EoU was rated independently by two experts (Drollinger-Vetter & Lipowsky, 2006). This procedure led to sufficient rater reliability for six of the nine items. For these six items, the generalizability coefficient (G) for relative decisions amounted over 0.65. For one of the nine items, the coefficient was lower than 0.58. For this reason, they brought about a consensus decision for those elements on which they had not reached an agreement. Because the ratings of the nine EoU must not necessarily correlate with each other, they computed the sum score of the occurrence of the nine elements. The mean of the sum scores was *M* = 22.45 (*SD* = 3.51), and the range varied between 14 and 27.

#### Scale “quality of modes of representation”

The EoU can be treated in different representational modes. The quality of each of the four modes of representation (formal, verbal, iconic, and enactive) was assessed separately on a four-point scale ranging from 1 (= low) to 4 (= high). For each class, an overall assessment was given. The inter-rater reliability was assessed by computing the value of G for relative decisions for any of the four items. The coefficients ranged between 0.66 and 0.85. The mean of the scale was *M* = 2.84 (*SD* = 0.60) and Cronbach’s alpha was *α* = 0.73.

#### Scale “structural clarity of content”

The scale “structural clarity” was based on four items (Appendix), which were rated between 1 (low) and 4 (high). The inter-rater reliability was assessed by computing the value of G for relative decisions for each of the four items. The coefficients ranged between 0.72 and 0.84. The mean of the scale was *M* = 2.70 (*SD* = 0.65) and Cronbach’s alpha was *α* = 0.88.

#### Interrelations of the three dimensions

Since the content of the three subject-specific dimensions overlapped and the coding of the EoU formed the basis for the other two dimensions, “quality of modes of representation” and “structural clarity of content”, the three dimensions might be inter-related. In fact, the correlations were high and significant (0.74**, 0.75**, 0.83**, Appendix, Table A3). An exploratory factor analysis (principal component analysis) showed that the three dimensions loaded on one factor. This superordinate factor can explain 84.6% of the total variance of all three items. The reliability of the scale comprising the three dimensions was *α* = 0.89. For further analyses, we used the mean of this scale, which we called “subject-specific quality of the Pythagorean unit.” It represents the occurrence, the quality, and the linking of EoU and the forms of representation.

### Analysis methods

For RQs 1a and b, the expert ratings were correlated with the aggregated perceptions of the students at the class level. To exclude the possibility of the correlations being influenced by class composition, we controlled for the mean mathematics achievement (T1) and the mean mathematics-related interest (T1) of the class.

To analyze the relation between the subject-specific quality of the Pythagorean unit rated by the experts and the students’ *individual* perceptions of their own learning process and comprehension and the general comprehension orientation of the teacher (RQs 2a, b), we used multilevel analyses. In addition, the prediction of achievement gains by individual student perceptions was analyzed through multilevel modeling (RQ 3) using HLM 6.04 (Raudenbush et al., 1993). All variables included in the analyses were grand-mean centered on level 1 (student) and grand-mean centered on level 2 (class) (cf. Lüdtke et al., 2009). Based on previous research (cf. Herbert et al., 2022), the students’ individual perceptions were assumed to be influenced by student characteristics (e.g., ability and interest) as well as factors on class level (e.g., mathematics performance of the class). Therefore, the mean achievement of the class in the Pythagoras pretest (T2) was included as a control variable in Models 1 and 2 (Table 2). For Model 3 (Table 2), which was used to analyze the long period of the whole school year, the mean achievement in the general mathematics test (T1) was controlled for. At the student level, we controlled for mathematics achievement (T2 or T1), interest in mathematics, and general cognitive ability.

## Results

Regarding RQs 1a and b, Table 1 shows that the raters’ assessments on subject-specific instructional quality were moderately aligned with the students’ (class-level) perceptions of their own learning processes, their own comprehension of the content as recorded immediately after the Pythagoras unit but also with the perception of the general comprehension orientation of the mathematics teacher, which was recorded at the end of the school year.

**Table 1:** Correlations between subject-specific quality of instruction (observer ratings) and students’ perceptions (partial correlations controlling for mathematical achievement and math-related interest, T1)

	Students: own learning process (Pyth)	Students: own understanding (Pyth)	Students: teacher’s overall comprehension orientation (T4)
Expert ratings: Quality (Pyth)	0.51**	0.47**	0.39*

*N* = 36 classes ** *p* < . 01

For RQs 2a and b, we analyzed whether individual student perceptions can be predicted by the expert ratings of subject-specific quality of instruction (Table 2). In Models 1 and 2, the focus was on students’ perceived learning and understanding related to the three Pythagoras lessons, which were also rated by the experts (RQ 2a). From a long-term perspective, the focus in Model 3 was on the individual perception of the teacher’s comprehension orientation in general, which was recorded at the end of the school year (RQ 2b). According to the results, the expert ratings of subject-specific instructional quality are reflected in the differences in students’ individual perceptions of their own learning processes and understanding after controlling for student and class characteristics. The regression weights for the subject-specific instructional quality are β = 0.16 in Model 1 and β = 0.12 in Model 2. For the overall understanding orientation of the teacher, this relation only emerged as a trend (β = 0.20, *p* < 0.10).

**Table 2:** Predicting student perceptions of their own learning and understanding and of teachers’ comprehension orientation

	Model 1: Student: own learning process (Pyth)		Model 2: Student: own understanding (Pyth)		Model 3: Student: teacher’s overall comprehension orientation (T4)	
	β	*SE*	β	*SE*	β	*SE*
*Class-level variables*						
Mean achievement (Pyth pretest)	−0.12*	(0.05)	−0.18**	(0.06)	−	
Mean achievement (math t1)	−		−		*nss*	
Expert ratings: Quality (Pyth)	0.16**	(0.06)	0.12*	(0.05)	*nss*	
*Individual-level variables*						
Achievement (Pyth pretest)	0.13**	(0.05)	0.16**	(0.05)	−	
Achievement (math t1)	−		−		*nss*	
Math-related interest	0.25**	(0.04)	0.27**	(0.04)	0.11**	(0.04)
General cognitive abilities	*nss*		0.15**	(0.04)	*nss*	

*Note.* β standardized HLM regression weight, *SE* standard error

** *p* < . 01, * *p* < 0.05, *nss* not statistically significant

Models 1 and 2 indicate that the students’ mathematics-related interest and individual mathematics performance are important predictors for their perceptions, while class performance has a negative effect. Model 1 explained 16.74% of the variance in students’ perceptions of their learning process; in Model 2, the included variables explained 20.23% of the variance in students’ perceptions of their own comprehension.

Regarding RQ 3, Models 4 to 6 (Table 3) were used to examine the extent to which individual student perceptions can predict the development of mathematics achievement in the Pythagoras teaching unit or over the entire school year. This is the case for the perception of one’s own learning and understanding as well as the overall comprehension orientation of the teacher when controlling for other important influencing factors. However, the regression weights β = 0.09, β = 0.11, and β = 0.06 are low.

**Table 3:** Predicting students’ achievement gains

	Model 4: Achievement: Pyth Post-test		Model 5: Achievement: Pyth posttest		Model 6: Achievement (end of school year)	
	β	*SE*	β	*SE*	β	*SE*
*Class-level variables*						
Mean achievement (Pyth pretest)	0.31**	(0.06)	0.33**	(0.07)	−	
Mean achievement (math t1)	−		−		*nss*	
*Individual-level variables*						
Achievement (Pyth pretest)	0.15**	(0.03)	0.15**	(0.03)	−	
Achievement (math t1)	−		−		0.22**	(0.05)
Math-related interest	0.08*	(0.03)	0.07**	(0.03)	0.13**	(0.03)
General cognitive abilities	0.22**	(0.03)	0.20**	(0.03)	0.26**	(0.04)
Student: own learning process (Pyth)	0.09**	(0.03)	−		−	
Student: own understanding (Pyth)	−		0.11**	(0.03)	−	
Student: teacher’s overall comprehension orientation	−		−		0.06*	(0.03)

*Note.* β standardized HLM regression weight, *SE* standard error

** *p* < . 01, * *p* < 0.05, *nss* not statistically significant

## Discussion

The starting point for our study were findings from previously conducted teaching quality research, which showed that the convergent and prognostic validity of student assessments of cognitive activation was rather limited. This concerns a quality dimension that is expected to play a central role in comprehension-oriented teaching. In this research, cognitive activation was usually recorded in the context of the TBD model as a generic quality dimension. Against this background, our study aimed to contribute to the investigation of the validity of student perceptions, considering subject-related characteristics of teaching quality and an opportunity-use model.

In summary, our results show a remarkable correlation between observer and student judgments at the *class level* when the observers’ judgments refer to subject- specific quality features of understanding-oriented teaching and the students’ judgments refer to the related learning processes and their understanding. Observer ratings are also reflected in *individual* student ratings of their own understanding and learning. Moreover, individual student ratings of their own learning processes and comprehension predict learning success when important learning prerequisites (interest, general cognitive ability, and prior knowledge) are being controlled.

### Teaching quality as a co-production of teachers and students: the role of opportunity-use models in assessing subject-specific characteristics of instructional quality

#### Convergent validity of students’ perceptions

According to opportunity-use models, the extent to which teaching promotes and supports students’ subject-related understanding and learning processes does not result directly and necessarily from the quality of the learning opportunities in terms of teacher actions and tasks but rather from an interaction between these learning opportunities and the quality of how these are used. Therefore, from a theoretical viewpoint, teaching quality should be seen as a co-production of teachers and students (Cai et al., 2020; Fend, 1998; Reusser & Pauli, 2010; Vieluf & Klieme, 2023). From a methodological viewpoint, the question arises as to how the quality of use can be adequately measured, especially for characteristics that relate to students’ understanding and learning processes. While observers can directly assess quality features of the offer, based on the features of teacher action, they have limited access to the students’ mental processes and thus to the use of the offering. One promising possibility was explored by Prediger et al. (2023), who recorded active participation in class discussions as an indicator of usage. One problem could be that students may be cognitively active but not verbally involved in the interaction. Therefore, it makes sense to include the students’ perspective as well (Vieluf, 2022).

##### Comparison of student and expert perceptions at the class level

Previous studies have recorded student and observer assessments and, in some cases, compared them with each other, primarily for characteristics of the learning opportunities offered by the teacher. In contrast to these studies, which found rather low correlations between expert and student assessments of cognitive activation (Fauth et al., 2020), our experts focused on subject-specific learning opportunities and the students on their use. The substantial correlations between the two perspectives can be explained, on the one hand, by the fact that by focusing on “subject-specific quality”, our experts specifically assessed the aspects of instruction that students need to build a robust understanding of the content (Pythagorean theorem), while the students assessed the related learning and understanding processes. It is undoubtedly easier for students to adequately assess their own learning and understanding of the subject matter than the subject-specific quality of instruction since they do not need subject didactic expertise to do so. The only requirement is that they can realistically assess their understanding and learning. On the other hand, it can be assumed that the observers did not base their assessment of subject-specific quality exclusively on features of teacher behavior or task setting but also drew conclusions about students’ processes of understanding. The quality assessments were related to clearly defined and content-standardized analysis units, which was not the case in most previous studies. This could also have contributed to a higher level of agreement between the expert and student assessments. Overall, the students’ and observers’ judgments in our study can be viewed as complementary sources for assessing the subject-specific quality of the instructional unit, understood as an interplay of offer and use.

Compared to the correlations between the student and observer perspectives related to the Pythagorean unit, the correlation is somewhat lower but still significant at the class level when students assess the general comprehension orientation of a mathematics teacher. Note that the student and observer judgments refer to different time periods and were collected at different times. While the observer judgments refer to the three Pythagorean lessons, the students retrospectively judged their mathematics teacher’s general comprehension orientation at the end of the school year. As mentioned earlier, previous studies have mostly found little or no correlation between the perspectives on the cognitive aspects of learning support (see 2.2). In contrast, our observers did not assess generic but subject-specific quality characteristics.

##### Predicting individual student assessments: the role of student and class characteristics

While individual students’ perceptions of learning and understanding can be predicted by observer ratings of subject-specific instructional quality (Table 2), the best predictor of students’ individual self-assessments is not the quality of teaching as assessed by the experts but the interest of the students. This finding is consistent with the research that has shown that students’ perceptions of instruction are influenced by individual student characteristics (cf. Herbert et al., 2022; Wang & Eccles, 2016). This might also be the case for students’ perceptions of their own cognitive processes and comprehension (Merk et al., 2021).

The finding that students’ self-assessments of their learning and comprehension are also influenced by individual prior knowledge can be explained by learning psychology, which stresses the active, constructive, and cumulative character of learning processes (e.g. Aebli, 1983; Chi & Wylie, 2014; Reusser, 2006). The more and better interlinked the content-related prior knowledge is, the more successful the understanding and learning will be. The negative effect of mean prior knowledge at the class level is rather surprising. Two explanations are possible. First, it could be the result of the reference group effect (Marsh, 2005), as it can be assumed that comparisons with the class play a role when assessing one’s own understanding and learning processes. The self-assessment may be more critical in comparison with a more capable group than in comparison with a less capable group, regardless of how well the learning content was understood and how successful one’s own learning process was. A second possible explanation is that teachers adapt their teaching to the performance of the class and increase the expectations of the students depending on the level of the learning group. From this viewpoint, the negative effect could result from being confronted (in the more efficient classes) with higher expectations, more difficult tasks to solve, and more demanding teacher questions. Both explanations may also apply simultaneously.

#### Prognostic validity

Students’ self-assessment of their learning and understanding also predicts their learning success. However, the effects of student perceptions are comparatively small. In contrast, the effects of the general cognitive ability as well as of prior knowledge on student and class level are, as expected, greater in terms of the short-term learning progress in the Pythagorean unit, which is equally positive at both levels.

Overall, previous research results have ascribed some prognostic validity to students’ perceptions of instructional characteristics in predicting learning outcomes, with inconsistent findings for cognitive activation (cf. 2.2.2). In contrast to this research, the students in our case assessed their own learning processes and understanding and not the quality of teaching in terms of teacher behavior or the tasks. In this context, the question arises as to what extent learners can realistically rate their own learning processes and comprehension. Our results indicate that this is the case, at least to a certain degree, even if the relations are rather weak and the understanding was only recorded with a single item. This finding is consistent with previous research on self-perception of learning and comprehension processes (e.g. Lingel et al., 2019). For example, Nuthall and Alton-Lee (1990) showed, using data from student interviews, that even young students can describe their understanding and learning processes in a differentiated and precise manner.

### Limitations, future research, and conclusions

Several features of our study require a discussion of its limitations. First, the analyses relate to a relatively small sample of classes and teachers that is not representative. Since participating in this study required considerable effort from the teachers and their willingness to have their lessons videotaped several times, it can be assumed that the participating teachers were a positive selection of particularly motivated and perhaps particularly competent teachers. Therefore, the results are not generalizable to all teachers and students in lower secondary schools in the two participating countries. Second, the data were collected in the 2002/2003 school year, around 20 years ago, as part of the “Pythagoras study” (Klieme et al., 2009). This multi-faceted study generated a very rich database of test, survey, and video data, which still contains potential for further analysis from new perspectives. This can be achieved using a wide range of available video codes and ratings and by capitalizing on previous analyses. However, the age of the data raises the question of the validity of such analyses and their findings. The development of digital media over the last 20 years, among other things, has not only changed the world in which young people live, but has presumably also changed the design of mathematics lessons, for example the use of media. Nevertheless, we still consider our analyses and their results to be justifiable today. This is particularly because media use is a surface characteristic that says little about the quality of teaching, whereas our analyses focused on the deep structural quality of the lessons (cf. 2.1). If we were to analyze our lessons in terms of their design (e.g. methods, media) and compare them with today's lessons on the introduction of the Pythagorean theorem, we would probably find different patterns or frequencies and thus describe the design of lessons then and now differently. In contrast, the analyses presented here are not aimed at describing mathematics education (then or now) per se, but rather at investigating deep structure dimensions and relationships of teaching–learning quality (Klieme et al., 2009). The extent to which such relationships are influenced by changes in the design of lessons in terms of surface features such as methods and media is an open question that cannot be answered based on our analyses. In any case, it would be interesting and important to replicate our results based on current and representative data sets.

A third limitation is that the students’ own perceived understanding was only recorded with a single item. It is therefore important that this variable is considered together with the other variable on the self-assessment of learning processes and understanding, which is based on a scale and with which it correlates, as expected (see Table A1, Appendix). It should also be noted that, in contrast to the students' perceptions of their own cognitive activity and their understanding, the students' perceptions of the teacher's comprehension orientation were only surveyed at the end of the school year.

Finally, since our analyses focused not only on subject-specific but also on content-specific characteristics of teaching quality, the results apply only to this content (i.e. the introduction of Pythagoras' theorem). It would be interesting to replicate our analyses with another rating instrument that measures the subject-specific characteristics of teaching quality. As mentioned in 2.2.1, numerous instruments have now been developed that cannot be listed here (overview e.g. in Praetorius & Charalambous, 2018; Schlesinger & Jentsch, 2016). Instruments based on the TBDs (e.g. the instrument developed for TEDS-Instruct, cf. Schlesinger et al., 2018) would be particularly interesting, as TBDs were also the basis for the Pythagoras study (Lipowsky et al., 2009) and are increasingly gaining international acceptance as a suitable framework for recording student assessments (cf. Herbert et al., 2022; Senden et al., 2023). One question of growing interest is whether and how the idea of EoU can also be transferred to other content in mathematics education (Korntreff & Prediger, 2022).

In the context of opportunity-use models of teaching and learning, it could also be interesting to have the students rate not only their use of the provided learning opportunities (in the present case: quality of the understanding and learning processes) but also the characteristics of the offer itself. However, due to the subject-specific knowledge discussed above, the question arises as to what extent such a rating would be possible and useful. In the present study, we refrained from doing so because we found assessments of students’ own learning more promising as an indicator of their use of the learning opportunities than complementary judgments to the observer ratings. It was also important that the students could complete the questionnaire as spontaneously as possible and with as little time as possible at the end of the teaching unit.

In summary, our study contributes to a more nuanced picture of the potential of student perspective in assessing the deep structural quality of teaching. This is done by showing that the perspectives of students (by looking at the quality of their learning processes and understanding) and observers (by looking at the subject-didactic quality of the learning opportunities provided by the teachers) are two complementary sides of the same coin. Both are necessary to obtain a more complete picture of what is happening in the classroom.

## Supplementary Information

Below is the link to the electronic supplementary material.

Supplementary file 1 Appendix, with more detailed information on the measurement instruments (4.3 to 4.5), is available online. (PDF 1329 KB)

## Footnotes

1 Since the Pythagorean theorem is part of the ninth-grade curriculum in Germany and the eighth-grade curriculum in Switzerland, we included German ninth-grade classes and eighth-grade classes from the German-speaking part of Switzerland.

2 Four classes were excluded from the analyses (2 teachers cancelled the study before it was completed; in one class, the students’ data were incomplete; and in another class, the teacher did not teach the content as required).
