# Supplementary material for: Capturing the subject-specific quality of mathematics instruction: How do expert judgments relate to students’ assessments of the quality of their own learning and understanding?
Source: ZDM. 2024 Mar 20;56(5):893–905. doi: 10.1007/s11858-024-01561-3 (PMC11469968; doi:10.1007/s11858-024-01561-3)
Supplement: Supplementary file 1 — Appendix, with more detailed information on the measurement instruments (4.3 to 4.5), is available online. (PDF 1329 KB) [file 11858_2024_1561_MOESM1_ESM.pdf]

Supplementary Information

To: “Capturing the subject-specific quality of mathematics instruction: How do expert judgments relate to students' assessments of the quality of their own learning and understanding?” (Pauli, Lipowsky & Reusser)

Online Appendix: Documentation on the survey and observation instruments

Note: All Items translated from German.

Details to 4.3: Measures of students’ perceptions

“Students’ perceptions of their learning process” (scale)

In these math lessons, …

1. I was able to follow the teacher's explanations.
2. I understood the teacher's explanations.
3. I was able to grasp the different solution steps.
4. I was able to follow the discussion of the tasks well.

Response format: (1) «disagree completely» to (4) «agree completely»

“Students’ perceptions of their own attained Pythagoras-related understanding” (single-item question)

How well have you understood the content that you went through?
(1) not at all, (2) hardly at all, (3) rather not, (4) rather, (5) fairly well, (6) very well

“Students’ perceptions of the overall comprehension orientation of the math teacher”

Four bipolar Items, response range between 6 and 1:

It is important to our math teacher ...

Very (6)	fairly (5)	rather (4)	Rather (3)	fairly (2)	very (1)
1. that we understand the content in class			that we can give answers as quickly as possible		
2. that we understand the path to the solution when solving tasks			that we arrive at the solution as quickly as possible when solving tasks		
3. that we check for ourselves how well we understand the content			Our math teacher doesn't care if we check whether we understand the material ourselves		
4. that we have understood a topic before we start the next one			that we progress quickly and complete the topics given in the math book		

Correlations between the three different perceptions

Table A1: Correlations between the different student perceptions on the student level

	Students: own understanding (Pyth, t3)	Students: teachers’ overall comprehension orientation (t4)
Students: own learning process (Pyth, t3)	.66**	.22**
Students: own understanding (Pyth, t3)		.11**

N >730 students; ** p <. 01

Calculation of ICC(1) and ICC(2)

Table A2: ICC(1) and ICC(2) for scales of students' perceptions

	Students: own learning process (Pyth, t3)	Students: own understanding (Pyth, t3)	Students: teachers' overall comprehension orientation (t4)
ICC(1)	.11	.09	.15
ICC(2)	.72	.69	.79

Details to 4.4: Students' mathematics-related interest

Students' mathematics-related interest (8-item-scale, recorded at t1, beginning of the school year)

1. Mathematics is exciting.
2. I would never do math voluntarily. (-)
3. Mathematics is very important to me personally.
4. I don't enjoy math. (-)
5. Mathematics is very useful to me.
6. To be honest, I don't care about math. (-)
7. I like math.
8. Mathematics is boring. (-)

Response format: (1) «disagree completely» to (4) «agree completely»
(-): Negatively worded items that have been reversed

Details to 4.5: Expert ratings of domain-specific instructional quality

Unit of analysis

The unit of analysis comprises all phases in which concepts or rules are introduced (e.g. developed, discovered based on a problem), theorems are formulated, and proofs are carried out. Teaching phases in which the already known theorem was practiced and applied with the help of exercises were not included. An analysis of these practice phases with a focus on the cognitive level of tasks worked on has been published elsewhere (Drollinger-Vetter et al., 2006).

Elements of understanding (EoU)

1. The core figure is a triangle.
2. The Pythagorean theorem is only true for *right-angled* triangles.
3. There are two different types of sides in a right-angled triangle.
4. It is important to distinguish these two types of sides.
5. The Pythagorean theorem makes statements about the length of the sides in a *right-angled* triangle.
6. The Pythagorean theorem is formulated focusing on the sides of the right-angled triangle (side aspect). By applying the Pythagorean theorem, the length of the sides in a right-angled triangle can be calculated.
7. The Pythagorean theorem is formulated focusing on the area aspect (e.g. In any right-angled triangle the sum of the areas of the squares above the legs are equal to the area of the square whose side is the hypotenuse).
8. There is a connection between the formulation via the sides aspect and via the area aspect.
9. By formulation the Pythagorean theorem it is made an explicit distinction between the presupposition and the assertion of the statement, e.g. using if-then-statements.

Quality of modes of representations

Four ratings (one each for iconic, formal, verbal and enactive representation) were given on a four-point scale from 1 (low) to 4 (high). "Quality" includes both comprehensibility/clarity and correctness. In all forms of representation, the right angle in the triangle must be recognizable or named. Further, specific quality criteria were formulated for each form of representation (Drollinger-Vetter & Lipowsky, 2006, pp. 193/194).

The four ratings were combined to form an overarching rating.

Structural clarity of content (4-item rating scale from (1) low to (4) high level of quality):

1. Quality of the elements of understanding (overall assessment)
2. The representation and visualization tools are used in such a way that they promote the construction of structure.
3. The links between the forms of representation contribute to the development of understanding.
4. The coherence of Pythagoras' theorem and its proof is comprehensible.

Response format: "strongly disagree" (1), " rather disagree" (2), "rather agree" (3), "strongly agree" (4)

Interrelations of the three dimensions

Table A3: Correlations between the dimensions of the domain-specific instructional quality

	quality of modes of representation	structural clarity of content
occurrence of conceptual elements (EoU)	.75**	.74**
quality of modes of representation		.83**

N=36 classes ** *p* <. 01
